# Identification of Tumor Antigens and Immune Landscape in Glioblastoma for mRNA Vaccine Development

**DOI:** 10.3389/fgene.2021.701065

**Published:** 2021-08-30

**Authors:** Liguo Ye, Long Wang, Ji’an Yang, Ping Hu, Chunyu Zhang, Shi’ao Tong, Zhennan Liu, Daofeng Tian

**Affiliations:** Department of Neurosurgery, Renmin Hospital of Wuhan University, Wuhan, China

**Keywords:** glioblastoma, tumor antigens, immunophenotyping, cancer vaccination, bioinformatics

## Abstract

**Background:** Clinical benefits from standard therapies against glioblastoma (GBM) are limited in part due to the intrinsic radio- and chemo-resistance. As an essential part of tumor immunotherapy for adjunct, therapeutic tumor vaccines have been effective against multiple solid cancers, while their efficacy against GBM remains undefined. Therefore, this study aims to find the possible tumor antigens of GBM and identify the suitable population for cancer vaccination through immunophenotyping.

**Method:** The genomic and responding clinical data of 169 GBM samples and five normal brain samples were obtained from The Cancer Genome Atlas (TCGA). The mRNA_seq data of 940 normal brain tissue were downloaded from Genotype-Tissue Expression (GTEx). Potential GBM mRNA antigens were screened out by differential expression, copy number variant (CNV), and mutation analysis. K-M survival and Cox analysis were carried out to investigate the prognostic association of potential tumor antigens. Tumor Immune Estimation Resource (TIMER) was used to explore the association between the antigens and tumor immune infiltrating cells (TIICs). Immunophenotyping of 169 samples was performed through consensus clustering based on the abundance of 22 kinds of immune cells. The characteristics of the tumor immune microenvironment (TIME) in each cluster were explored through single-sample gene set enrichment analysis based on 29 kinds of immune-related hallmarks and pathways. Weighted gene co-expression network analysis (WGCNA) was performed to cluster the genes related to immune subtypes. Finally, pathway enrichment analyses were performed to annotate the potential function of modules screened through WGCNA.

**Results:** Two potential tumor antigens selected were significantly positively associated with the antigen-presenting immune cells (APCs) in GBM. Furthermore, the expression of antigens was verified at the protein level by Immunohistochemistry. Two robust immune subtypes, immune subtype 1 (IS1) and immune subtype 2 (IS2), representing immune status “immune inhibition” and “immune inflamed”, respectively, had distinct clinical outcomes in GBM.

**Conclusion:** ARPC1B and HK3 were potential mRNA antigens for developing GBM mRNA vaccination, and the patients in IS2 were considered the most suitable population for vaccination in GBM.

## Introduction

Grade IV glioblastoma (GBM), known as the most lethal type of brain cancer, was highly aggressive, and the survival rate of patients with GBM was extremely low (Li et al., 2021). The median survival time of patients was 12–15 months, and the 5-year survival rate was less than 5% (Cristofaro et al., 2020). Surgical resection combined with radiotherapy and chemotherapy were still the primary methods for GBM therapy, while the diffusive and invasive feature of GBM makes the complete removal of the tumor by conventional treatment strategy nearly impossible (Ferber et al., 2017). Despite adjuvant radiotherapy and chemotherapy, the clinical outcome of GBM patients remains miserable with a high recurrence rate for the resistance of GBM to chemotherapies (Quick et al., 2010). As a result, the need is urgent for developing novel strategies to improve the therapeutic condition of GBM.

Nowadays, cancer immunology and immunotherapy have come a long way and made progress on many solid tumors (Ribas and Wolchok, 2018). Through improving the immunity of the body, immunotherapy produces an immune response to tumors (Zhu and Chen, 2019). With immune checkpoint inhibitors (ICIs) targeting programed cell death protein 1 (PD-1) and its ligand 1 (PD-L1) had achieved effect on malignant tumors, the mRNA-cancer vaccine therapy has become a hotspot in cancer immunotherapy (Pardi et al., 2018). Tumor vaccines can lead to the initiation of a systemic anti-tumor immune response and restore the intrinsic ability of the immune system to recognize tumor cells, then eventually eliminate occult and metastatic tumors (Ogi and Aruga, 2013; Pardi et al., 2018). First of all, the identification of tumor-associated antigens (TAAs) is a primary requirement for a successful tumor vaccine development (Zirlik et al., 2006). The forms of antigens for a tumor vaccine could be a peptide, tumor cell, dendritic cell, DNA, or RNA type (Park et al., 2019). However, compared with the first four types, mRNA vaccines are highly feasible for targeting tumor-specific antigens and promising immunotherapy strategies in clinical treatment (Mockey et al., 2007; Kaitsuka and Tomizawa, 2015; Nguyen et al., 2019). Moreover, several studies have proved the effectiveness of the possibility of mRNA tumor vaccines in clinical trials (Kübler et al., 2015; Hong et al., 2016). In addition, the effect of the tumor vaccine depends on the characteristics of the tumor immune microenvironment (TIME; Geng et al., 2020; Ning et al., 2020). For example, promoting the infiltration of killing tumor cells, including CD8^+^ T cells, into the tumor is necessary to boost the effectiveness of cancer vaccines (Geng et al., 2020). Instead, a low level of immune infiltration or immunosuppressive microenvironment of tumor tissues may significantly reduce the effect of immunotherapy (Ning et al., 2020). Therefore, it is also an essential part of the development of tumor vaccine treatment to identify the suitable population according to the function of TIME.

Considering that almost no research reported the development of GBM-related mRNA tumor vaccine so far, genes with mutation and copy number variant (CNV) associated with a poor survival and positively correlated to the infiltration of antigen-presenting cells (APCs) were identified TAAs for developing GBM mRNA vaccines. Based on the clustering of immune subtypes, two robust immune subtypes were identified based on the features of TIME in each subtype. We then screened three functional modules that are closely related to the subtypes through weighted gene co-expression network analysis (WGCNA). These findings provided a theoretical basis for developing mRNA cancer vaccines against GBM. It also described an immune landscape and identified a candidate population for mRNA cancer vaccination therapy.

## Methods and Data

### Public Data Obtaining and Processing

The normalized mRNA_seq data and corresponding clinical information of 169 GBM and 5 normal tissues were acquired from The Cancer Genome Atlas (TCGA). The gene expression data of 940 normal brain tissue samples were downloaded from the Genotype-Tissue Expression (GTEx) project. Then, the mRNA data in TCGA and GTEx were merged and normalized as one cohort by the R package “limma” (Reble et al., 2017). The data of genes with somatic mutations in the VarScan2 (Reble et al., 2017) platform and information of genes with CNV of 532 normal samples and 628 GBM samples were acquired from TCGA.

“Bonferroni” method was applied to identify the genes with different copy number variations (CNVs) in the normal and GBM tissues (The adjustable *p*-value less than 0.05 was considered statistically significant). R package “maftools” was used to identify the mutant genes in GBM. Overexpressed genes in the tumor were identified in the merged cohort by the “limma” package based on the criterion: Log [fold change (FC)] > 1 and adjustable *p*-value < 0.05.

### ESTIMATE Analysis

By the “estimate” algorithm, the immune infiltration level, including the stromal and immune scores of each GBM sample, was calculated. According to the median value of the stromal and immune scores, respectively, the samples were divided into high and low score groups, genes differentially expressed in the low and high score groups were screened by the “limma” package and defined as immune-related differentially expressed genes (IRDEGs).

### Prognosis Analysis

Overall and disease-free survival (DFS) analysis was performed based on Kaplan–Meier curves to explore the prognostic value of potential GBM antigens. Log-rank *P*-value < 0.05 was considered significant. In addition, univariate Cox regression was also used to screen out the prognosis-related antigens in GBM (*p* < 0.05 was considered significant).

### Tumor Immune Estimation Resource Analysis

Tumor Immune Estimation Resource (TIMER; Li et al., 2017) database visualized the association between the abundance of tumor-infiltrating immune cells (TIICs) and prognosis-related antigens in GBM. Considering the purity adjustment of GBM, Spearman’s correlation analysis was used to investigate the relationship between the potential mRNA antigens and APCs, including macrophages, B cells, and dendritic cells.

### Human Protein Atlas Analysis for APCs-Related Antigens

According to The Human Protein Atlas (HPA) database,^1^ immunohistochemical staining for potential GBM antigens was done to investigate the protein level among normal brain and high-grade glioma [World Health Organization (WHO) IV] tissues.

### Identification of Immune Subtypes

Consensus clustering was performed by The “ConsensusClusterPlus” package to identify robust immune clusters based on the comparison of the abundance of 22 kinds of tumor-infiltrating immune cells (TIICs) evaluated through the “cibersort” (Chen et al., 2018) algorithm among 169 GBM samples. Then, K-M survival analysis was used to explore the difference in the overall survival (OS) among different subtypes. Meanwhile, the clinical and TIICs profiles were investigated to compare the clinical and TIME characteristics among immune subtypes.

### The Single-Sample Gene Sets Enrichment Analysis

A total of 29 immune signatures (He et al., 2018) representing diverse immune cell types, functions, and pathways were quantified for their enrichment degrees within the respective GBM samples using single-sample gene set enrichment analysis (ssGSEA) by the “GSVA” package (Hänzelmann et al., 2013).

### Differential Analysis of ICDs, TMB, and ICPs

The expression level of immunogenic cell death modulators (ICDs) obtained from previous studies (Huang et al., 2021) and vital immune check points (Cimen Bozkus et al., 2019), PDCD1 (PD1), CD274 (PD-L1), and CTLA4, were compared among different immune subtypes by Pairwise *t*-tests. Furthermore, we compared the distribution of tumor mutational burden (TMB) between the immune groups.

### Weighted Gene Co-expression Network Analysis

By the differential expressed analysis of 19,645 differential expressed genes (DEGs) among the normal and GBM tissues in immune subtypes, 712 genes were screened out for further WGCNA by the “WGCNA” package (Langfelder and Horvath, 2008). Highly variable genes of the HPC population were detected, and gene modules were examined by dynamic hybrid cut. The correlation between module genes and immune subtypes was investigated. Gene Ontology (GO) and Kyoto Encyclopedia of Genes and Genomes (KEGG) analysis were performed by the “clusterProfiler” package (Yu et al., 2012) to annotate the functions of the module genes that are closely correlated to the immune subtypes.

## Results

### Identification of Six Genes as Potential Antigens

**FIGURE 1:**
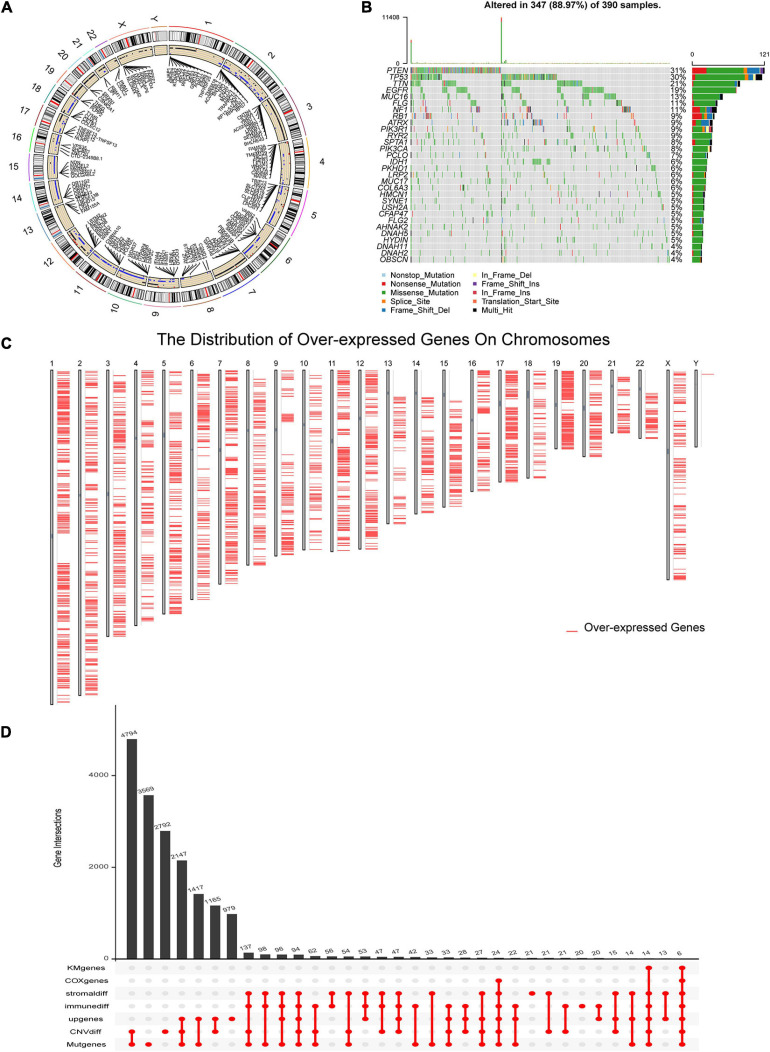
Identification of potential glioblastoma (GBM) vaccine mRNA antigens. **(A)** The chromosomal distribution of the genes with copy number variant (CNV) in GBM. **(B)** Waterfall plot of the distribution of the top 30 mutant genes in GBM. **(C)** Overexpressed genes and the location of the corresponding chromosome according to Gene Expression Profiling Interactive Analysis (GEPIA) dataset. **(D)** An Upset plot displays the intersections of genes screened under different conditions. GBM, glioblastoma; geneMut, mutant genes; CNVdiff, genes with different copy number variation; upgenes, upregulated genes in GBM; immunediff, differentially expressed genes among low and high immune score groups; stromaldiff, differentially expressed genes among low and high stromal score groups; COXgenes, the genes with *P*-value less than 0.05 in univariate Cox analysis; and KMgenes, the genes with *P*-value less than 0.05 in K-M survival analysis.

To identify the potential antigens of GBM, we first screened out 11,528 genes with a significantly different CNV and 12,684 mutant genes in the GBM samples, respectively. The circle plot (Figure 1A) showed the genes with CNV and the corresponding chromosome positions. Mutant situations of the top 30 genes with the highest mutation frequency were exhibited using a waterfall plot (Figure 1B). Considering that the effect of immunotherapy is related to the level of immune infiltration in tumors, 920 and 870 genes were found to be associated with the immune and stromal scores, respectively. Since TAAs are considered overexpressed in tumors, we identified 12,015 overexpressed genes in GBM compared to normal brain tissues. Overexpressed genes and the corresponding chromosome positions are shown in Figure 1C. The prognostic value of potential tumor antigens in GBM is also an indicator of therapeutic efficacy. Subsequently, univariate Cox regression and K-M survival analysis were performed to identify the prognosis-related genes in GBM. Six genes, including ADAMTS14, HK3, ARPC1B, LTBP2 PTX3, and PLAUR, were screened out for further analysis, the numbers and intersections of the identified genes from different methods were visualized using an upset plot (Figure 1D).

### The Prognostic Value of Six Potential Antigens

**FIGURE 2:**
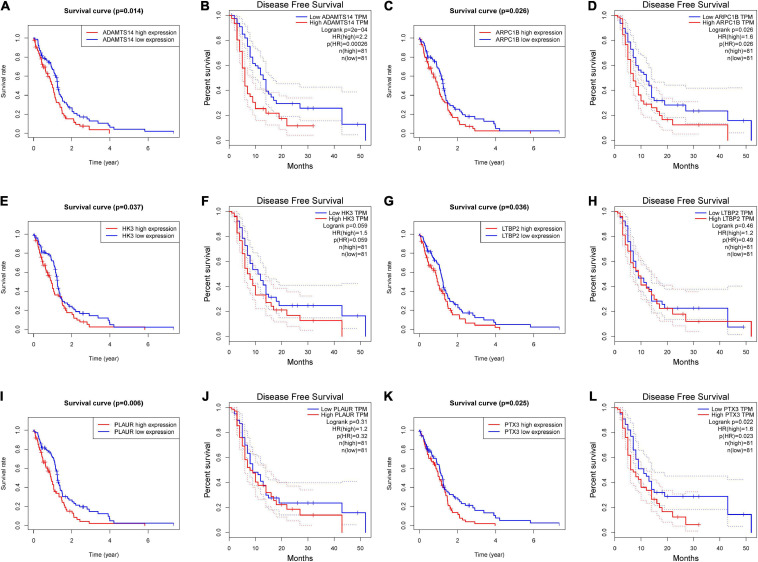
The prognostic value of six potential GBM mRNA antigens. K-M curves showed the overall survival (OS) and disease-free survival (DFS) of patients with GBM in the different expression levels of **(A,B)** ADAMTS14, **(C,D)** ARPC1B, **(E,F)** HK3, **(G,H)** LTBP2, **(I,J)** PLAUR, and **(K,L)** PTX3. Genes with *P*-value < 0.05 were considered significantly correlated to the prognosis of GBM. DFS, disease-free survival.

We further demonstrated the survival analysis results of these six potential antigens, including overall and DFS analysis. According to Figures 2A–L, ADAMTS14, ARPC1B, and PTX3 were correlated to the OS and DFS in GBM, while HK3, LTBP2, and PLAUR were only associated with the OS. We found that the upregulation of these genes above was both associated with a worse prognostic outcome in GBM.

### Correlation Between Six Potential Antigens and APCs

**FIGURE 3:**
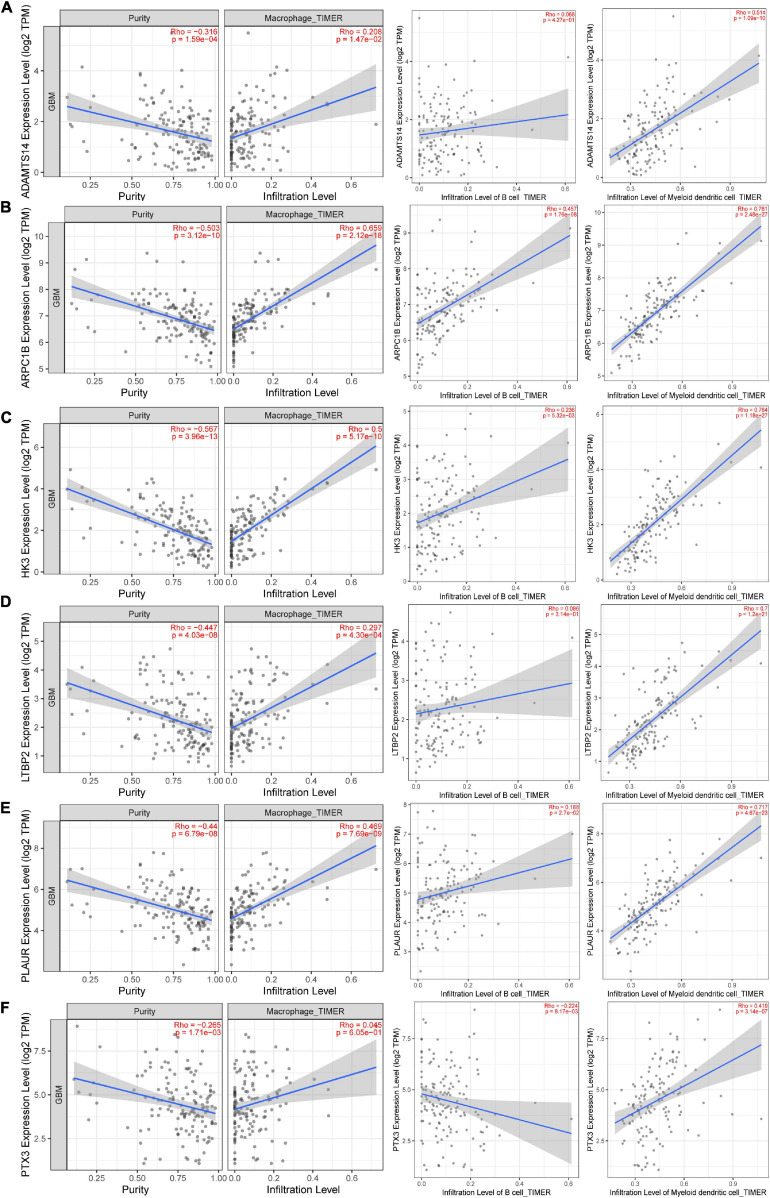
The association between six potential mRNA antigens and antigen-presenting immune cells (APCs). According to the Tumor Immune Estimation Resource (TIMER) 2.0 database, the correlation between tumor purity, the infiltration level of APCs (Macrophages, B cells, and myeloid dendritic cells), and the expression level (Log2 TPM) of **(A)** ADAMTS14, **(B)** ARPC1B, **(C)** HK3, **(D)** LTBP2, **(E)** PLAUR, and **(F)** PTX3. APCs, antigen-presenting cells.

Antigen-presenting cells play a significant role in the onset of protective immunity (Park et al., 2018). Dendritic cells are central to initiating, regulating, and maintaining immune responses while also playing an essential role in inducing anti-tumor immune responses (Wculek et al., 2020). The role of B cells as APCs has been extensively studied, mainly about activating memory T cells and initiating APCs (Popi et al., 2016). Based on the TIMER algorithm, the infiltration level of three kinds of APCs is significantly positively correlated with the expression level of ARPC1B, HK3, and PLAUR, as shown in Figures 3A–F. These findings suggest that the three identified tumor antigens may trigger a better immune response than others. Therefore, ARPC1B, HK3, and PLAUR were the more promising TAAs for developing mRNA vaccines against GBM.

### Verification of Identified TAAs at the Protein Level

**FIGURE 4:**
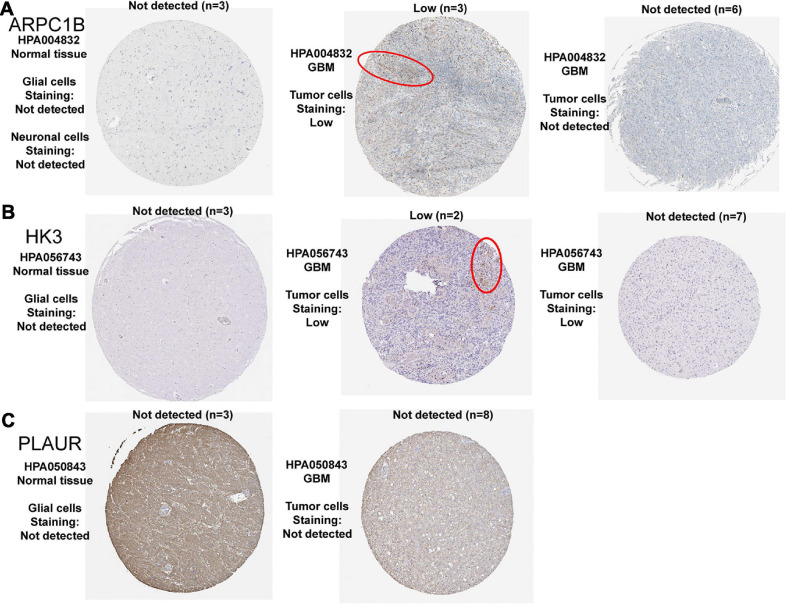
Representative IHC images of three prognosis-related antigens in normal brain tissues and GBM tissues. **(A)** ARPC1B, **(B)** HK3, and **(C)** PLAUR.

After the screening above, we have obtained three potential tumor antigens. We then explored the expression of the three candidates at the protein level. The results of Immunohistochemistry in the HPA database showed that the expression of ARPC1B and HK3 proteins could be detected in GBM, while there was no significant expression of ARPC1B and HK3 proteins in normal brain tissues (Figures 4A,B). However, the expression of PLAUR protein was not significantly detected in both normal brain and GBM tissues (Figure 4C). So far, we have identified the expression of ARPC1B and HK3 at both mRNA and protein levels in GBM. These results confirmed the possibility of the two candidates as TAAs for developing GBM mRNA vaccination, including ARPC1B and HK3.

### Identification of Immune Subtypes of GBM

**FIGURE 5:**
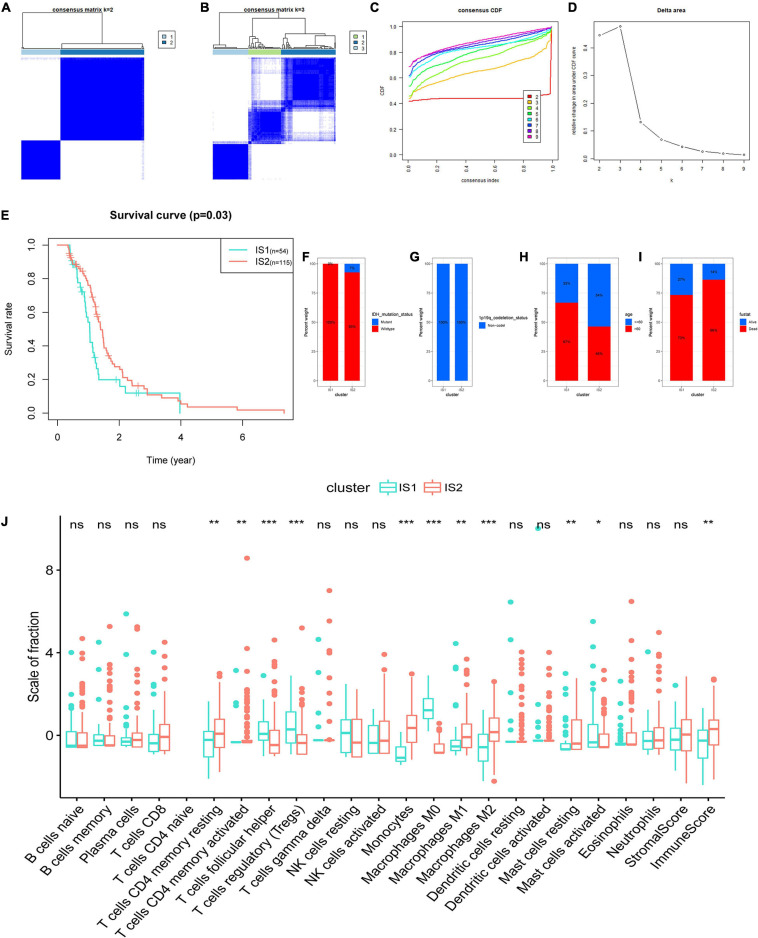
Identification of immune subtypes of GBM based on the consensus clustering of the abundance of 22 kinds of tumor immune infiltrating cells (TIICs). **(A)** Consensus clustering matrix of 169 The Cancer Genome Atlas (TCGA)-GBM samples for *k* = 2 and **(B)** *k* = 3. **(C)** Consensus clustering CDF for *k* = 2 to *k* = 9. **(D)** Relative change in area under CDF curve for *k* = 2 to *k* = 9. **(E)** Survival analysis between the OS and two subtypes. Distribution ratio of **(F)** IDH mutation status, **(G)** 1p19q co-deletion status, **(H)** age group (cut off: 60 years old), and **(I)** survival status among immune subtype 1 (IS1)-immune subtype 2 (IS2) in TCGA-GBM. **(J)** The difference analysis of the abundance of immune cells and the stromal and immune scores in IS1 and IS2. Fustat: survival status. ***p < 0.001, **p < 0.01, *p < 0.05, ns: not significant.

The tumor microenvironment (TME) of GBM has a certain heterogeneity. Therefore, it is of great significance to distinguish groups with different immune microenvironment characteristics through immunotyping, which is also necessary for selecting patients suitable for tumor vaccine treatment. Based on the abundance of 22 kinds of TIICs in GBM, the subtype clustering appeared stable when *k* = 2 (Figure 5A), while when *k* = 3, the boundaries between the data began less straightforward (Figure 5B). In addition, immune typing data are categorized into two groups which were defined as immune subtype 1 (IS1) and immune subtype 2 (IS2) according to the corresponding cumulative distribution function and function delta area of the *K* value (Figures 5C,D). Survival analysis in Figure 5E shows a significant difference between different subtypes, in which the samples in IS1 had the worse OS. We further investigated the percent weight of proportion for different clinicopathological subtypes in IS1 and IS2, respectively. The proportion of IDH mutation status and 1q19q co-deletion status are shown in Figures 5F,G. These results indicated that GBM had the consistency of the status of IDH mutation and 1p19q co-deletion, and the two clinical factors were unsuitable for further differentiation of GBM. In IS1, there were more patients with age of more than 60 years and had poor clinical outcomes compared to IS2 (Figures 5H,I). We then compared the fraction of TIICs and immune infiltration levels among IS1 and IS2 (Figure 5J). The abundance of M2, M1 macrophages, Monocytes, and CD4 T cells of the GBM samples were significantly higher in patients of IS2. While in IS1, M0 macrophages, regulatory T cells (Tregs), and follicular helper T cells were the main components compared to IS2. In addition, samples in IS1 had higher immune scores. Overall, the TIICs of IS1 mainly consist of differentiated macrophages and monocytes, and CD4 T cells. In contrast, IS2 is a subtype of undifferentiated macrophages and suppressive immune microenvironment, promoting the immune escaping of tumor.

### Immune Microenvironment Characterization in Immune Subtypes

**FIGURE 6:**
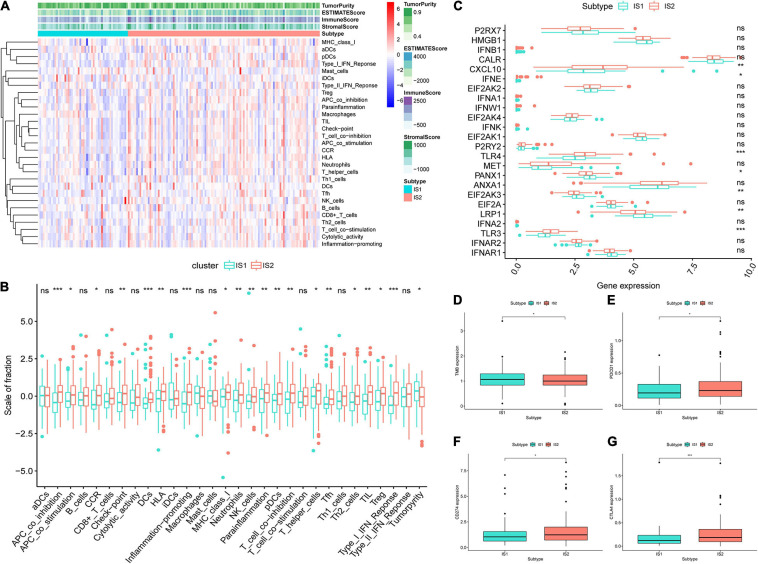
Features of tumor immune microenvironment (TIME) in different immune subtypes. **(A)** Based on the results of single-sample gene-set enrichment analysis (ssGSEA) of 29 immune-related hallmarks and pathways in GBM samples, heatmap showed the different levels of tumor purity, ESTIMATE score, immune score and stromal score, and the distribution of enrichment scores (ESs) of each sample in IS1 and IS2. The darker the color, the greater the absolute value of the score. **(B)** The difference analysis of ES of each sample changes among IS2 and IS2 was shown in the boxplots. **(C)** Different expression levels of immune checkpoint (ICP) genes in IS1 and IS2. **(D)** The difference analysis of tumor mutational burden (TMB) between IS1 and IS2. Difference analysis of **(E)** PDCD1, **(F)** CD274, and **(G)** CTLA4 among two subtypes. ***p < 0.001, **p < 0.01, *p < 0.05, ns: not significant.

The ssGSEA score could be employed for quantifying the activities or abundances of the immune signatures in different immune subtypes. In most cases, the enrichment scores (ESs) in IS2 were higher than that in the IS1 group, as shown in Figure 6A. The difference analysis of the ES values between IS1 and IS2 indicated that the samples in IS2 had significantly higher ESs and lower levels of tumor purity than in the IS1 (Figure 6B). Given the significance of immune checkpoints (ICPs) and ICDs in tumor immunity, we subsequently investigated the expression level of 24 kinds of ICDs in different subgroups and found that 7 ICPs were differentially expressed among two immune subtypes (Figure 6C). CXCl10, IFNE, TLR4, and TLR3 were upregulated in IS2, while PANX1, EIF2AK3, and LRP1 were overexpressed in IS1. In addition, samples in IS1 had a significantly higher TMB than in IS2 (Figure 6D). Moreover, CTLA4, PDCD1 (PD-1), and CD274 (PD-L1) had a higher expression level in IS2 (*p* < 0.05, Figures 6E–G). Compared to samples in IS1, GBM samples in IS2 had a stronger association with immunity, higher fraction of antigen-presentation cells (monocytes and macrophages), and higher expression levels of ICPs. Obviously, GBM patients in the IS2 subtype are more suitable for tumor vaccine treatment.

### Weighted Gene Co-expression Network Analysis of Characteristic Genes for GBM Immunotyping

**FIGURE 7:**
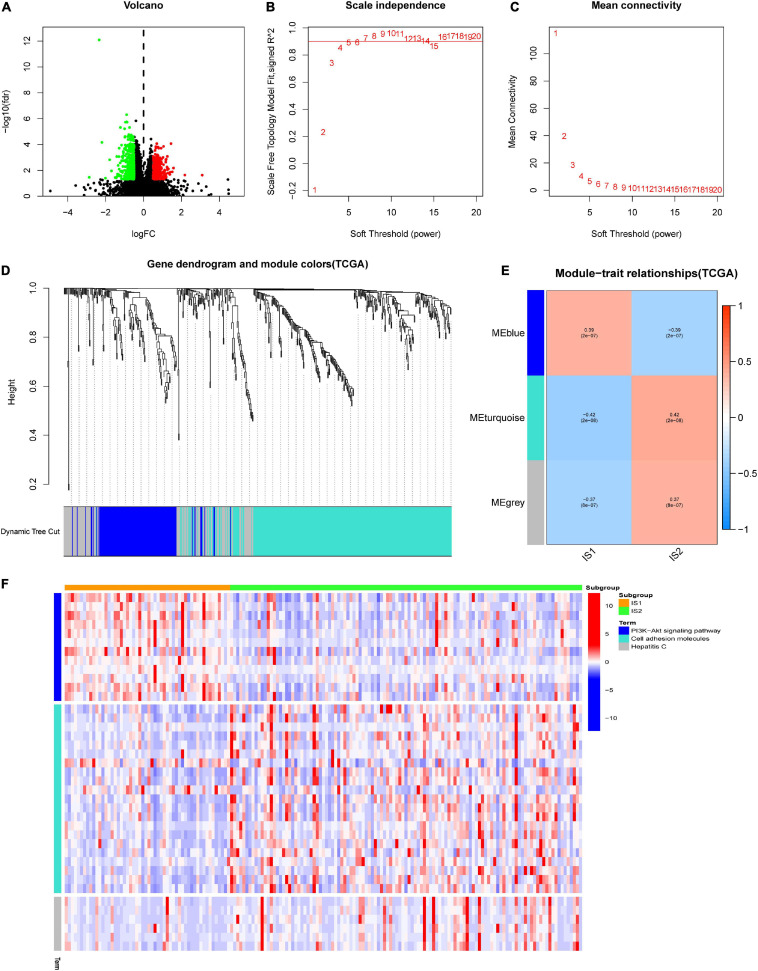
Weighted gene co-expression network analysis (WGCNA) of differential expressed genes (DEGs) between different immune subtypes in TCGA-GBM. **(A)** The volcano plot showed the differentially expressed genes among IS1 and IS2. The log∣FC ∣ > 1 and adjusted *P*-value < 0.05 were considered significant. The red dot represents the upregulated gene in IS2. Instead, the green dot represents the upregulated gene in IS1. The black dots mean no significant difference among the two groups. **(B,C)** Scale-free fit index and mean connectivity for various soft-thresholding powers (β). **(D)** DEGs were clustered using hierarchical clustering with a dynamic tree cut and merged based on a dissimilarity measure (1-TOM). **(E)** Relationship analysis between immune subtypes and modules. Color on the left represents a module, and red represents positive correlation, blue represents negative correlation, the darker the color, the stronger the correlation, and the value in brackets under the correlation coefficient is the calculated *p*-value. **(F)** Heatmap showed the expression level of different Kyoto Encyclopedia of Genes and Genomes (KEGG) terms involved DEGs of blue, turquoise, and gray module (MEgray) in IS1 and IS2, heat map colors correspond to the level of mRNA expression as indicated in the color range.

It is meant to identify the biomarkers related to each immune subtype to determine the subtype in which the patient is most likely to be. First, we identify 712 genes that are differentially expressed among IS1 and IS2, as shown in the volcano plot (Figure 7A). Subsequently, we clustered these DEGs by the WGCNA method, and selected five as the soft-thresholding power based on the scale-free fit index and the mean connectivity (Figures 7B,C). The colors of the dendrogram branches indicate different gene clusters, whereas the upper dendrogram shows the sample clustering (Figure 7D). Three modules were screened out according to the relationship between the modules and immune subtypes. According to the correlation coefficient and *p*-value (Figure 7E), gray module (MEgray) and turquoise module (MEturquoise) were negatively correlated to IS1 (MEgray: rho: −0.37, *p* < 0.05, MEturquoise: rho: −0.42, *p* < 0.05), while blue module (MEblue) was positively correlated to IS1 (rho: 0.39, *p* < 0.05). Figure 7F shows the KEGG terms enrichment analysis for the module genes, the genes of MEblue, which were overexpressed in IS1, were mainly involved in the P13/AKT pathway, and genes in MEturquoise upregulated in IS2 were significantly related to the terms of Cell adhesion molecules, and genes of MEgray positively correlated to IS2 mainly participated in Hepatitis C.

## Discussion

Tumor-associated antigens are significantly overexpressed in cancer compared to normal cells (Wagner et al., 2018). Antigen level is an important factor influencing the magnitude of the immune response. Nowadays, advances in next-generation sequencing (NGS), bioinformatics, and peptidomics have enabled the identification of non-synonymous mutations and other alterations of the cancer cell genome (intron retention, indels, frameshifts, etc.), emerging as neo-antigens and resulting in the development of personalized vaccines (Esprit et al., 2020). Neo-antigens could be recognized as non-self-epitopes and thereby enhance the immune reactivity against tumor cells (Cohen et al., 2015). In addition, CNV refers to a segment of DNA (Valsesia et al., 2013), CNVs are the most common genetic alteration in cancers, and CNV burden is associated with the rates of recurrence and death in multiple neoplasms (Hieronymus et al., 2018). Therefore, we considered the biomarkers with mutation possibility and upregulated expression in GBM as potential TAAs, which were more easily recognized by the immune system and more conducive to promoting the response of patients to a tumor mRNA vaccine (van Rooij et al., 2013).

Moreover, the degree of immune infiltration can largely affect the effect of immunotherapy (Liu et al., 2020), and a number of studies have demonstrated that the TME, particularly the tumor stromal cells, contribute to the malignant behavior of human gliomas (Zirlik et al., 2006). We have to take it as an essential indicator.

So far, this is the first report on the bioinformatics of the development for GBM vaccination. This study identified two potential prognosis-related TAAs, ARPC1B, and HK3, correlated to the immune infiltration level, screened out from overexpressed genes with mutation and CNV in GBM, and validated their expression at protein level by immunohistochemical staining. Moreover, the positive association of the two TAAs with APCs further confirmed their effectiveness and feasibility as antigens for GBM mRNA vaccines. ARPC1B may function as a p41 subunit of the human Arp2/3 complex that has been implicated in the control of actin polymerization in cells (Abella et al., 2016). The overactivation of the Arp2/3 complex generally promotes cancer progression (Molinie and Gautreau, 2018). In addition, ARPC1B is also essential for maintaining CTL cytotoxicity, and it could control the cell surface levels of TCR, CD8, and GLUT1 via its role in retromer and WASH-mediated recycling (Randzavola et al., 2019). However, the role of ARPC1B functions in GBM has not been systematically studied yet. As a potential GBM antigen, targeting ARPC1B in GBM may be of great significance to promote T-cell activation and the tumor-killing function of TIICs.

In addition, HK3 encodes hexokinase 3, which could phosphorylate glucose to produce glucose-6-phosphate, the first step in most glucose metabolism pathways (Rijksen et al., 1982). Research had found that the expression of HK3 in tumor tissues may be related to T cell activation and anti-tumor immunity (Tuo et al., 2020). As a cancer-related gene, HK3 plays a vital role in the TIME, so it may be feasible to consider it a TAA for GBM.

Tumor immune cell infiltration is a vital component of the TIME. The value of different immune cells obtained by the Cibersort algorithm could accurately reflect the landscape of TIICs (Chen et al., 2018). Subsequently, through the hierarchical consensus clustering based on the abundance of TIICs in the TCGA-GBM samples, two immune subtypes identified, IS1 and IS2, had distinct characteristics of TIME and clinical outcomes. Patients with GBM in IS1 had a worse OS than patients in IS1, suggesting that immunotype could be a prognostic biomarker for GBM. Furthermore, its prediction accuracy is better than conventional indicators such as IDH and 1p19q. IS2 had a higher immune infiltration level and better outcomes than IS1, a large part of the reason is that the fraction of immunosuppressive immune cells in IS1 is significantly higher than that in IS2. For example, a higher fraction of Tregs, a vital factor in tumor immune escape, concentrating on IS1 could suppress the function of immune effector cells through a variety of mechanisms (Bosch et al., 2004; Wang et al., 2015). Instead, M1 macrophages and memory resting CD4^+^ T cells, as the critical member for killing tumors and promoting immune response, were the main component in IS2 (Charoentong et al., 2017; Wei et al., 2019). Therefore, IS1 presented an immunosuppressive feature, while IS2 a pro-inflammatory type. It may explain the difference in prognosis between the two groups.

Moreover, samples in IS2 could better activate the immune response when treated with a tumor vaccine. In addition, the abundance of monocyte and macrophages in IS2 is significantly higher than that in IS1, which means that patients in IS2 may have a higher efficiency of tumor-specific or TAAs extraction, in turn triggering T-lymphocyte mediated anti-cancer immunity (Lee et al., 2020). However, the TIME of IS1 has a great anti-tumor potential for its higher composition of M0 macrophages and T-follicular helper (Tfh) cells. Undifferentiated macrophages (M0) could be induced to polarize into pro-inflammatory M1 macrophages by intrinsic molecular regulators and specific extrinsic environment conditions (Li et al., 2018), improving the effectiveness of immunotherapy for GBM patients. Through upregulating CXCR5, Tfh cells could home to the interface between the T cell and B cell regions of lymph nodes and interact with activated B cells through antigen presentation (Ansel et al., 2000).

Immune checkpoint inhibitors are a widely effective strategy of immunotherapies that block the inhibitory ICP pathways to reactivate immune responses against cancer (Cohen et al., 2019). In our study, the expression levels of PD-1, CD274, and CTLA4, the vital ICPs in many solid tumors, were significantly higher in IS2 than in IS1, which indicated that patients receiving ICIs therapies might achieve a better curative effect (Yang et al., 2021). Moreover, patients with GBM in IS2 may benefit more from the treatment strategy of combining with ICIs and mRNA tumor vaccine than in IS1. In general, immunophenotyping based on this study can well distinguish the prognosis of patients and the characteristics of the TIME. With better clinical outcomes, patients with GBM in IS2 were more suitable for mRNA tumor vaccinations. It is undeniable that patients in IS1 have a relative potential and possibility to benefit from cancer vaccine treatment, but it may overcome more difficulties.

In addition, DEGs among IS1 and IS2 were further clustered by WGCNA. Accordingly, the mRNA vaccine may not be suitable for patients with highly expressed genes aggregated into MEblues, negatively correlated to IS2. The functional annotation of the modules may provide a theoretical support for the follow-up study of the characteristic molecular markers of subtypes.

## Conclusion

In conclusion, ARPC1B and HK3 were considered the possible TAAs of GBM, and mRNA cancer vaccine therapy may be more beneficial for patients in IS2. In addition, this research provides a theoretical basis for the development of mRNA vaccine against GBM and provides a novel possible strategy of immunotherapy against GBM for patients in different immune subtypes.

## Data Availability Statement

The original contributions presented in the study are included in the article/supplementary material, further inquiries can be directed to the corresponding author.

## Author Contributions

LY and LW designed the study. LY and JY analyzed the data. LY and DT discussed the project. LY, PH, CZ, ZL, and ST drafted the manuscript. All authors read and approved the manuscript and agreed to be accountable for all aspects of the research in ensuring that the accuracy or integrity of any part of the work are appropriately investigated and resolved.

## Conflict of Interest

The authors declare that the research was conducted in the absence of any commercial or financial relationships that could be construed as a potential conflict of interest.

## Publisher’s Note

All claims expressed in this article are solely those of the authors and do not necessarily represent those of their affiliated organizations, or those of the publisher, the editors and the reviewers. Any product that may be evaluated in this article, or claim that may be made by its manufacturer, is not guaranteed or endorsed by the publisher.

## Footnotes

1
https://www.proteinatlas.org/

## Abbreviations

GBM: glioblastoma
WHO: World Health Organization
TIME: tumor immune microenvironment
TME: tumor microenvironment
TCGA: The Cancer Genome Atlas
GTEx: Genotype-Tissue Expression
IRDEGs: immune-related differentially expressed genes
CNVs: copy number variations
HPA: human protein atlas
GEPIA: Gene Expression Profiling Interactive Analysis
OS: overall survival
DFS: disease-free survival
TIMER: Tumor Immune Estimation Resource
TIICs: tumor-infiltrating immune cells
APCs: antigen-presenting cells
TAAs: tumor-associated antigens
ssGSEA: single-sample gene-set enrichment analysis
ICPs: Immune checkpoints
ICDs: immunogenic cell death modulators
GO: Gene Ontology
KEGG: Kyoto Encyclopedia of Genes and Genomes
Fig, figure. IS1: immune subtype 1
IS2: immune subtype 2
ICIs: immune checkpoint inhibitors
WGCNA: weight gene co-expression network analysis.
